# **BASEPROD: The Bardenas Semi-Desert Planetary Rover Dataset**

**DOI:** 10.1038/s41597-024-03881-1

**Published:** 2024-09-27

**Authors:** Levin Gerdes, Tim Wiese, Raúl Castilla Arquillo, Laura Bielenberg, Martin Azkarate, Hugo Leblond, Felix Wilting, Joaquín Ortega Cortés, Alberto Bernal, Santiago Palanco, Carlos Pérez del Pulgar

**Affiliations:** 1 Space Robotics Lab, Department of Systems Engineering and Automation, University of Málaga, Malaga, Spain; 2 Planetary Robotics Lab, Automation and Robotics Section, European Space Agency, Noordwijk, The Netherlands; 3 Télécom Nancy, Nancy, France; 4 Delft University of Technology, Delft, The Netherlands; 5 Applied Physics I, Faculty of Science, University of Málaga, Malaga, Spain

**Keywords:** Engineering, Aerospace engineering, Databases, Lasers, LEDs and light sources

## Abstract

Dataset acquisitions devised specifically for robotic planetary exploration are key for the advancement, evaluation, and validation of novel perception, localization, and navigation methods in representative environments. Originating in the Bardenas semi-desert in July 2023, the data presented in this Data Descriptor is primarily aimed at Martian exploration and contains relevant rover sensor data from approximately 1.7km of traverses, a high-resolution 3D map of the test area, laser-induced breakdown spectroscopy recordings of rock samples along the rover path, as well as local weather data. In addition to optical cameras and inertial sensors, the rover features a thermal camera and six force-torque sensors. This setup enables, for example, the study of future localization, mapping, and navigation techniques in unstructured terrains for improved Guidance, Navigation, and Control (GNC). The main features of this dataset are the combination of scientific and engineering instrument data, as well as the inclusion of the thermal camera and force-torque sensors in particular.

## Background & Summary

Datasets from analogue planetary exploration scenarios provide an effective means for algorithm development and verification prior to and in addition to more costly field testing campaigns. The collected data can serve for prototyping and validation of tasks related to Guidance, Navigation, and Control (GNC) such as perception and localization. Advancing the current state of the art for GNC components contributes to an increased scientific return of future planetary robotic missions^1^ as the rovers’ reach and safety improve.

While there are extensive and high-quality datasets to support automotive autonomy developments, their data does not cater to the different sets of problems encountered by planetary exploration. In contrast to planetary robotic exploration, the terrains encountered by the automotive industry are typically structured, dynamic, and cars use Global Navigation Satellite System (GNSS) navigation and a higher number of high-power sensors while driving at much higher speeds.

One example of datasets specifically conceived for planetary exploration scenarios is the Katwijk beach dataset^2^, which features artificial obstacles to support the development of obstacle detection and navigation algorithms. More representative, Lunar and Mars analogue sites were recorded later on, e.g., in Morocco^3,4^. The utility of such datasets is proven by their use in subsequent navigation method developments: Bahraini *et al*. used the Erfoud dataset^3^ to validate their approach for cooperative visual odometry^5^. The Katwijk beach dataset was used, among others, in the works of Boyu Kuang *et al*. to validate their rock segmentation algorithm^6^ and Federico Furlan *et al*. used both the Katwijk beach dataset and the Devon Island rover navigation dataset^7^ to assess and compare the performance of neural network-based detectors^8^ for planetary exploration.

To further explore and increase a rover’s situational awareness, the use of additional terrain information such as thermal data^9,10^, traction, and vibration^11^ have been proposed in the literature. These additional sensors can provide information about subsurface conditions and derive terramechanic properties which may in turn improve the planning for upcoming path segments. High-risk areas may more easily be identified and avoided.

While the topics focus on the engineering aspects of robotic exploration, the scientific goals often include the study of the ground and on finding features of interest in the surroundings. NASA has utilized two Laser Induced Breakdown Spectroscopy (LIBS) instruments, integrated in the so-called ChemCam^12^ and SuperCam^13^, on their Mars rovers Curiosity and Perseverance, respectively, to help identify the chemical composition of high-interest science targets at a distance of up to 7 m. The CNSA’s Zhurong rover incorporated another LIBS instrument, the MarSCoDe^14^.

LIBS information provides details about the chemical composition of the terrain along or even on the rover’s path. This could be used as a ground truth for the material and also to detect terrain features related to expected compaction, roughness, or salience, i.e. whether the sample represents materials which are uncommon in a given area and which are thereby of scientific interest. The use of this type of scientific instruments for rover navigation is pending investigation.

The dataset presented in this paper offers a combination of sensors that, according to the authors’ knowledge, is not encountered in other datasets, capturing data over a total distance of ca. 1.7 km and through varying terrains of the Bardenas semi-desert. In addition to stereo camera images, rover pose with absolute ground truth localization data, and a high-resolution drone map, the dataset contains thermal images of the terrain and Force-Torque (F/T) readings for all six sensors in the rover’s legs. One important property is that the rover traverses a range of surface types at various inclinations and speeds to experience changes in traction and conditions that induce a variety of resulting slip ratios. To complement the thermal camera readings, the dataset features recordings from a mobile weather station including irradiation, temperature, and humidity. Additionally, the spectra of rocks along the rover path were recorded via LIBS.

The dataset is available on ESA’s robotics dataset repository^15^, 10.57780/esa-xxd1ysw. Details about the field test preparation, lessons learned, and the base control station can be found in separate publications^16,17^.

## Methods

All data was recorded between the 20th and 23rd of July, 2023, in the Spanish Bardenas Reales semi-desert at 42°04′05.0″N, 1°30′10.1″W. The area belongs to Cabanillas, Navarra, Spain, and was chosen primarily for its resemblance to otherworldy planets and secondly for logistic considerations. The summer period was chosen to get a closer similarity to Martian landing site candidates while recording sensor data of the dried-up river bed, steep slopes, pebbles, and rock outcrops.

The rover was driven manually in direct teleoperation which allowed us to drive reactively and traverse varied and more challenging terrains. We could adapt the speed on the fly to climb steep slopes or record interesting terrains as we discovered them, which significantly reduced the planning effort and risk to the rover.

**Fig. 1:**
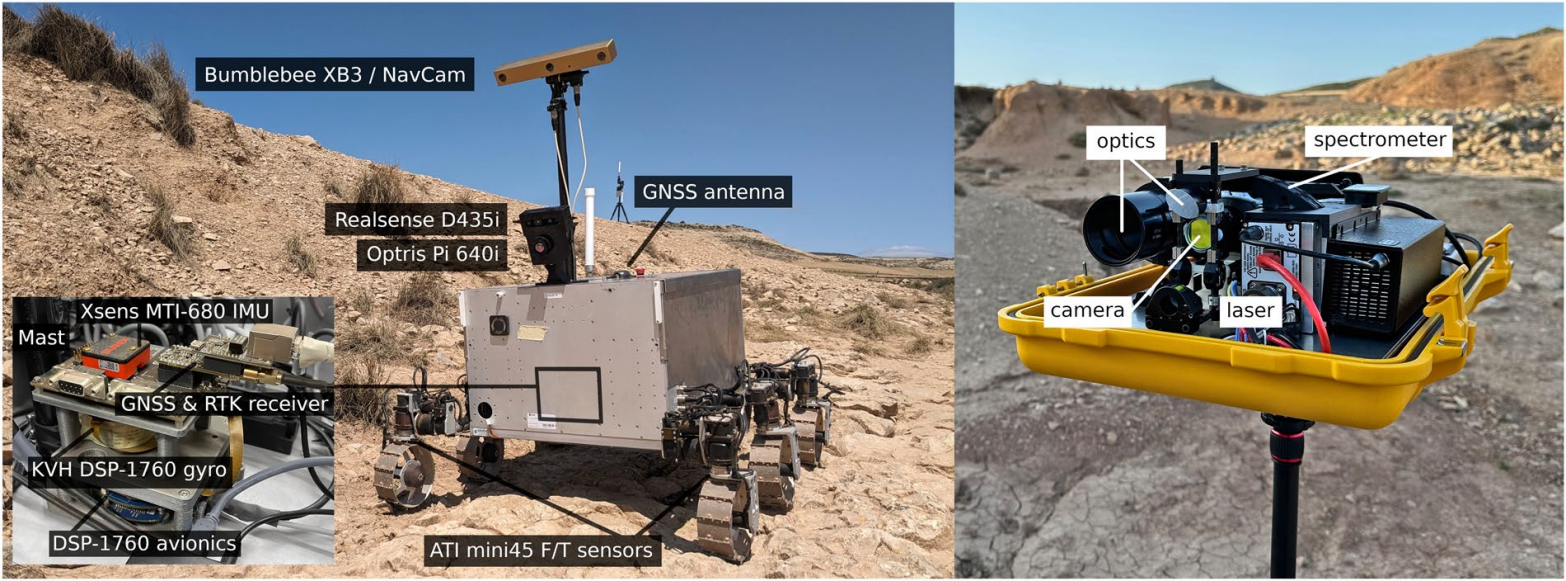
In addition to the commercial UAV and weather station sensors, the main data sources for the dataset are the MaRTA rover (left) and the portable LIBS instrument (right).

Not counting support equipment such as the rover operation control station, network equipment etc., the data collection was performed by four complementary systems: a half-scale model of the ExoMars Rosalind Franklin rover (see Fig. 1), an Unmanned Aerial Vehicle (UAV), a weather station, and a LIBS instrument (Fig. 1). All of these are explained in more detail in the following sections.

**Fig. 2:**
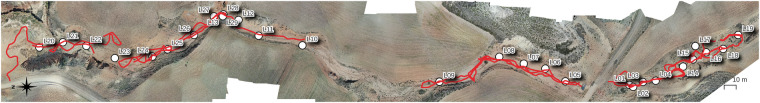
Locations of the LIBS measurements. The figure shows the traverse and LIBS GNSS data on the geo-referenced map.

Throughout the field campaign, the rover traversed a total of ca. 1.7 km, gathering ca. 450 GB of rosbag logs recorded in Robot Operating System 2 (ROS 2) Humble Hawksbill. Figure 2 shows an overview of the test area including the recorded rover traverses and LIBS measurement locations. All data has been exported from the rosbags and organized for use without ROS 2 or its dependencies. The weather station collected data continuously throughout each day of the campaign and a total of 28 sites were sampled with the LIBS instrument.

### Rover

The Martian Rover Testbed for Autonomy (MaRTA)^18^ is ESA’s half-scale model of the six-wheeled ExoMars Rosalind Franklin rover. It has a maximum driving speed of ca. 10 cm/s.

The following sensors are integrated in MaRTA:

- Xsens MTi-680 Inertial Measurement Unit (IMU) and GNSS receiver with external Real Time Kinematic (RTK) receiver: This sensor provides orientation and acceleration information as well as absolute GNSS rover positions.
- Realsense D435i RGB-D stereo camera: This camera combines color images and depth information. The integrated IMU data is omitted in favor of the above-mentioned Xsens MTi-680. The camera is mounted in the front center, just above the rover chassis, and tilted down 20° to observe the terrain ahead rather than the sky while still seeing the horizon.
- Bumblebee XB3 stereo camera: This color stereo camera has a large baseline of 24 cm between the left and right lens. Interested parties may use this camera to obtain depth information at further distances. The camera is located atop the mast-mounted Pan-and-Tilt Unit (PTU) and to be pointed at different distances. The camera is included to offer an alternative vantage point and a wider baseline compared to the Realsense camera. Including its stereo image pairs also allows for custom depth computations.
- Optris PI 640i thermal camera: This thermal camera is mounted just below the Realsense camera and captures the thermal signature of the terrain.
- KVH DSP-1760 Fiber Optic Gyro (FOG): This single-axis Fiber Optic Gyro records the rover’s yaw orientation. The sensor is used to augment the rover IMU’s orientation information.
- Six ATI mini45 (F/T) sensors (one at each rover wheel leg): These (F/T) sensors can be used by themselves or in combination with the IMU to obtain information about the terrain that is being traversed by the rover.

The exported data formats are explained in the Data Format section.

### Mapping drone

A high-resolution aerial map was created using the photogrammetry software Pix4D (https://www.pix4d.com/) and is based on images recorded by a DJI Mavic 2 pro UAV. We performed four drone flights to sweep the test area containing the rover traverses and LIBS measurements. All images taken during these flights contain GPS tags and for increased precision, we took reference coordinates of visually distinct targets on the ground. Using Pix4D, we then generated a high-resolution, geo-referenced 3D map of the environment including its texture.

### Weather station

We recorded ambient temperature, humidity, and pressure using a Bosch Sensortec BME280 sensor and a Davis Instruments SKU 6450 solar radiation sensor recorded the solar irradiance.

### Spectroscopy

The use of LIBS allows for the study of the terrain that the rover traverses. This study is done using a portable LIBS instrument that allows for the study of the terrain in a short period of time, enabling a range of experiments and measurements along the different rover routes.

The LIBS instrument combines an Nd:YAG laser (Viron, Quantel, 6 ns, 25 mJ at 532 nm, 20 Hz repetition rate) with a compact spectrometer (Avaspec Mini, Avantes B.V.). A set of custom-made lenses can focus the laser in a 2 to 6 m range and collect the plasma light back to the instrument. On impinging a surface, the laser pulse induces a plasma which emits light related to the elemental sample composition. A solid angle of the plasma emission is collected and a spectrum is registered and stored in the onboard computer per each laser pulse. In addition, a camera synced with the laser pulse captures the sample scene for the accurate location of each plasma on the sample when the instrument is subjected to vibration from wind or motion of the carrier platform^19^.

The data on the terrain in Bardenas was taken in different positions along the paths in places where the terrain denoted certain differences from its surroundings. The places where the measurements were taken can be seen in the Fig. 2.

## Data Records

**Fig. 3:**
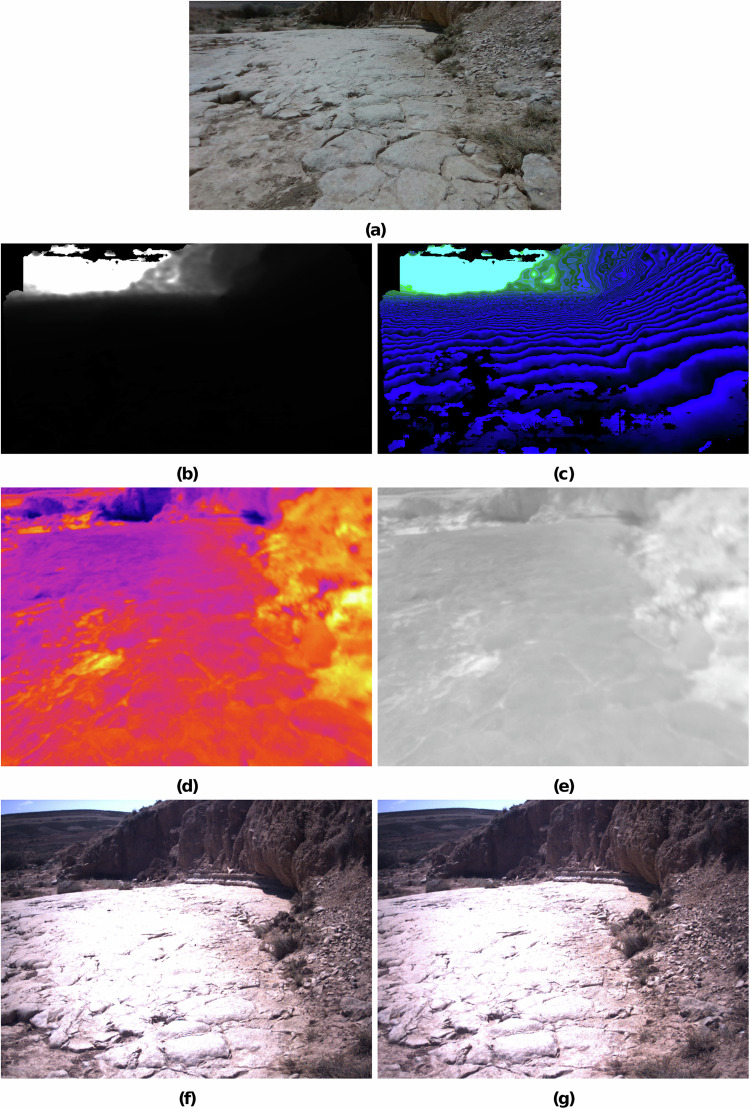
Samples of exported rover images: (**a**) Realsense color images, (**b**) Realsense 16-bit grayscale depth images, (**c**) Realsense depth images with RGB encoding, (**d**) thermal RGB (normalized to individual image value range), (**e**) thermal absolute values in the range of 10 to 50°C, (**f**) Bumblebee XB3 left, and (**g**) Bumblebee XB3 right.

**Fig. 4:**
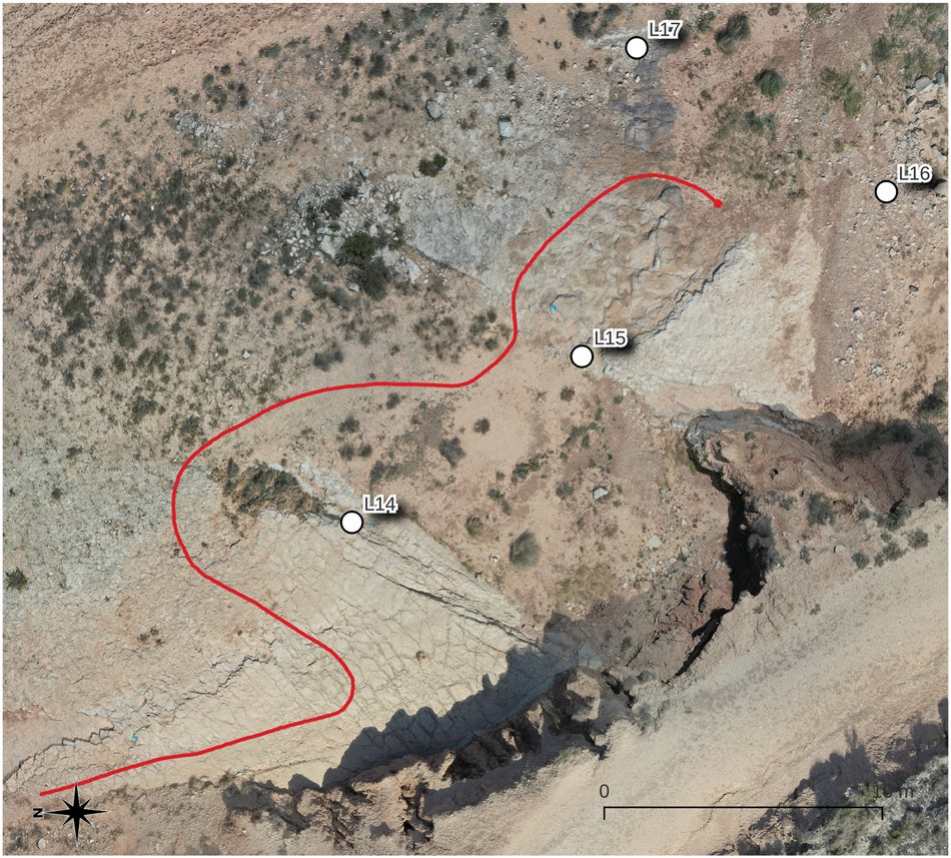
Sample traverse 2023-07-23 13-05-11. LIBS recordings are marked to facilitate orientation. Note the compass in the lower left corner: East is up and North is left.

The dataset is available at ESA’s robotics dataset repository^15^. A breakdown of the collected data can be seen in Table 1 and Fig. 3 shows exemplary exported images taken by the rover. The dataset contains 24 traverses varying in length from 6.85 to 202m, totaling ca. 1.7 km. Figure 4 shows a traversed path and LIBS recordings in the same area. Such overview images can be found in each traverse’s subdirectory. The traverses show different terrain characteristics based on which a user may decide to use one rather than another. Additional insight can be gained by viewing the provided traverse overview images mentioned above.

**Table 1:** Overview of the collected rover data, amounting to a total of ca. 450GB in MCAP rosbags.

Data	Rate [Hz]	Entries
Thermal images	1	36,000
Thermal RGB images	1	36,000
RS depth images	1	36,000
RS color images	1	36,000
Navcam images	1	62,000
(F/T) measurements	100	16,037,000
GNSS position	4	109,000
IMU	50	1,373,000
FOG	100	2,746,000
Transformation tree	18	480,000

The image topics were recorded at 1.2 to 1.4 Hz instead of the sensors’ maximum frequencies in the order of 30 Hz. This was considered a good compromise between data usability and storage. The table reports the lowest observed value and the number of entries is an approximation to give an idea of the data volume. The transformation tree is ROS’s representation of transformations between rover coordinate frames.

### Directory structure

**Fig. 5:**
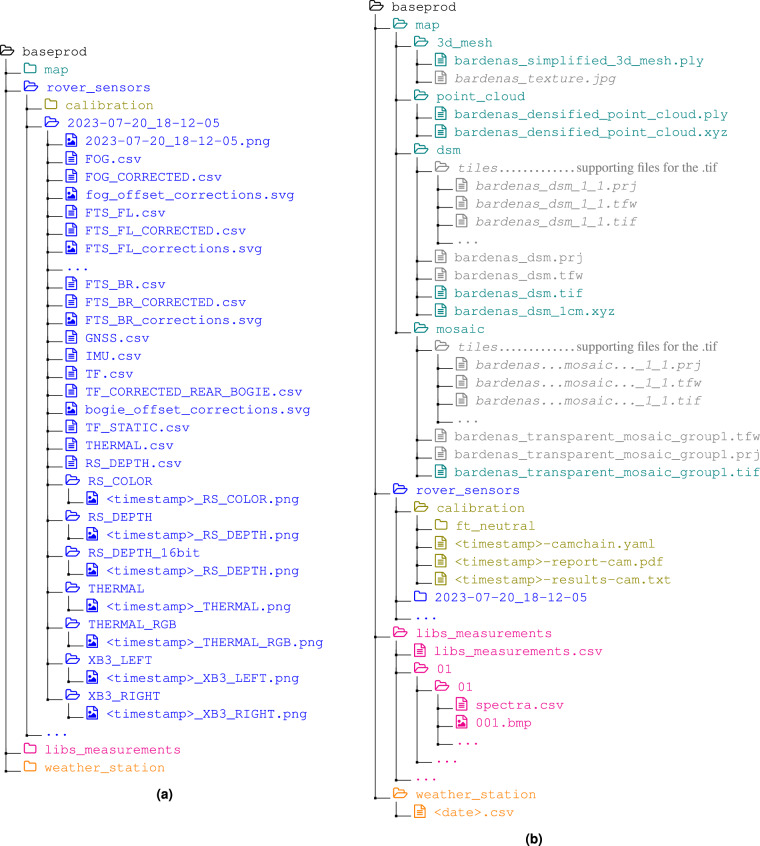
Directory tree showing (**a**) the available data for individual traverses and (**b**) the structure for map data, calibration files, LIBS measurements, and weather station recordings.

Figure 5 shows the directory structure. Exported images follow the naming scheme <timestamp>_<sensor>.png and reside in the sensor’s subdirectory of the individual traverses. Data which is exported as Comma-Separated Values (CSV) files are located directly in the respective traverse directory. LIBS data can be found in the libs_measurements directory. In this LIBS root directory, you will find a CSV listing each measurement’s coordinates and related filenames. The measurements are further sorted into directories named after the measurement location identifier and spectrum measurement number. Each of those leaf directories contains the measurement picture showing the measurement target, and each acquisition location directory contains a CSV with all measured spectra. Weather station data is located in the weather_station directory.

### Data formats

This section presents the different data formats used in the dataset^15^. The focus lies on using open data formats wherever possible. Additionally, the data contains convenience files that are useful for quick visual inspection of the data to find the most pertinent parts for a given use case. This is why we provide the thermal and depth data in three formats for example.

#### File types

- .png A common image format that allows lossless compression. This file type is chosen for exported rover camera images.
- .bmp Windows Bitmap files contain raw image data and can be displayed by all standard image viewers. The LIBS instrument spectrum images are saved in this format.
- .svg Scalable Vector Graphics (SVG) is a vector graphics format that is often used where rasterized images lose too much detail at higher zoom settings. We use this to export graphs that document the data. SVG files can be viewed with most standard image viewers or opened and modified with tools such as Inkscape (https://inkscape.org/).
- .csv CSV files are plain-text files containing tabular data. A line is a table row and columns are separated by commas. These files can be opened with any text editor or spreadsheet tool.
- .xyz, .ply, .tif These files contain the drone map information. .xyz files contain point clouds (with x, y, and z coordinates), Polygon File Format (PLY) files may contain point clouds or meshes and can be opened in 3D software such as MeshLab (https://www.meshlab.net/). Tagged Image File Format (TIF) is used for the geo-referenced, textured map and can be opened in Geographic Information System (GIS) software such as QGIS (https://qgis.org/).
- .tfw and .prj Supporting text files used by Pix4D to geo-reference raster images (https://support.pix4d.com/hc/en-us/articles/204174165-TFW-World-File-Values) and to specify the coordinate system (https://support.pix4d.com/hc/en-us/articles/202559979-How-to-obtain-or-create-a-prj-coordinate-system-syntax-file-PIX4Dmapper).
- .jpg A common image format that is used by Pix4D to export the simplified 3D mesh’s texture.
- .mcap MCAP (https://mcap.dev/) is the default storage format for ROS 2 logs/rosbags. Since the rover data was recorded with ROS 2 Humble Hawksbill (https://docs.ros.org/en/humble/index.html), the recommended way to read them is the use of ROS 2 or the Python package rosbags (https://pypi.org/project/rosbags/) (tested with version 0.9.16).

#### Conventions

All exported data uses UNIX timestamps in nanoseconds since 00:00:00 UTC on January 1st, 1970, as opposed to the ROS 2 convention of decimal seconds with nanoseconds. All data adheres to the International System of Units (SI) unless specified otherwise. Transformations follow the right-hand rule.

#### GNSS localization data

The GNSS data in GNSS.csv comprises all fields of the ROS 2 NavSatFix (https://docs.ros2.org/latest/api/sensor_msgs/msg/NavSatFix.html) message type plus converted location data. This means that the message contains the timestamp, RTK status (RTK fixed (status ≥ 0) or degraded accuracy (status < 0) and service (which satellites are being used)), latitude, longitude, altitude, position covariance matrix (3 × 3 matrix, which yields 9 covariance fields in row order in the CSV file), and the location in meters in the UTM grid as UTM_Easting and UTM_Northing. The UTM zone is 30T.

#### Force and torque measurements

The (F/T) measurements are exported in six CSV files per traverse; one for each of the six (F/T) sensors. The (F/T) sensors are identified by their mounting points on the rover, i.e., FTS_FL is the (F/T) sensor in the f ront l eft-hand side wheel assembly, FTS_CL is in the c enter l eft-hand side, and FTS_BR is in the b ack r ight-hand side.

- FTS_<ID>.csv These CSV contain a Timestamp field followed by three fields for the measured force in *x*, *y*, *z* direction respectively, and the measured torques, equally around *x*, *y*, and *z*.
- FTS_<ID>_CORRECTED.csv These CSV contain the same fields and timestamps as the corresponding FTS_<ID>.csv, but the force and torque values are updated according to the procedure discussed in the Technical Validation section, accounting for recorded calibration values when the rover is standing still.

#### Inertial Measurement Unit (IMU) and Fibre Optic Gyro (FOG)

Both IMU and FOG data contain the exported fields of the ROS 2 message of type IMU Message (https://docs.ros2.org/latest/api/sensor_msgs/msg/Imu.html) followed by rotation Euler angles in around *z*, *y*, and *x* in radians (in the order ZYX). Only parts of the message fields are relevant for each sensor though, which is why the exported formats differ. Note that the single-axis FOG measures the orientation around the *z* axis and no translational accelerations.

- IMU.csv The IMU data contains the timestamps, orientation quaternions split over four columns (*x*, *y*, *z*, and *w*), angular velocity [rad/s] around *x*, *y*, *z*, linear acceleration [m/s^2^] along the three axes, and finally the orientation in Euler angles [rad] along the three axes in ZYX order.
- FOG.csv The FOG only features the timestamps, orientation quaternions split over four columns (*x*, *y*, *z*, and *w*), angular velocities around the three axes as well as the orientation around the FOG’s *z* axis in radians. The latter is equivalent to the FOG’s heading estimate relative to its orientation at the start of the traverse.
- FOG_CORRECTED.csv The FOG records its heading relative to its starting orientation but it is not aligned to a cardinal direction. The corrected values are the raw FOG recordings plus an offset to ensure that the orientation of 0 corresponds to the rover facing East.

#### Realsense camera

We recorded two data streams from the Realsense camera, color images and depth images. Fig. 3a shows an example color image, and Fig. 3c shows the corresponding depth image. The Realsense depth information is aligned with the Realsense color images. The alignment is done by the manufacturer. The exported depth information is available in three formats.

- RS_COLOR directory 1280 × 720pixel RGB PNG images
- RS_DEPTH_16bit directory 848 × 480pixel 16-bit unsigned grayscale PNG images (Fig. 3b)
- RS_DEPTH directory 848 × 480pixel 24-bit RGB PNG images (Fig. 3c)
- RS_DEPTH.csv One CSV per traverse where each line contains one 848 × 480 depth frame in row-major order.

In all three cases, the depth is provided in millimeters. I.e., in the grayscale images, the intensity value is the depth in millimeters, and every cell in the CSV (except header and timestamp) is the depth of the corresponding pixel in millimeters, too. Some image libraries cannot handle 16-bit images well. Some convert them to 8-bit images, for example, dropping half the information. Hence, we also provide the depth encoded as RGB images, where the blue channel represents the least significant bits and the green channel represents the most significant bits while the red channel is not used.

We compute the green (*G*) and blue (*B*) values for depth *d* as in equations (1) and (2):

1 $$G=\lfloor \frac{d}{256}\rfloor $$

2 $$B=d\,{\rm{mod}}\,\,256$$

A depth of 300 mm at pixel (4, 5) at time *t* will thus be represented as a value 300 in the corresponding 16-bit grayscale image (in <t>_RS_DEPTH_16bit.png), as value 300 in column DATA_<4+5*848> and row with timestamp *t* in RS_DEPTH.csv, and as an RGB value of R=0,G=1,B=54 in the corresponding RGB image (in <t>_RS_DEPTH.png).

#### Optris thermal camera

Each thermal image contains 640 × 480 temperature entries. The data is saved in two formats within the rosbags: (1) a matrix of absolute measurements in°C and (2) a relative RGB representation. The thermal data is exported in three different formats, the first two of which are based on the same thermal_float data matrix, the third one being an export of the thermal_RGB image.

- THERMAL.csv A traverse’s THERMAL.csv contains all raw thermal scans of that traverse; One image per line in row-major-order preceded by its corresponding timestamp.
- THERMAL directory The THERMAL images represent the absolute measurements. The values inside these images are all normalized with the same limits of 10 to 50°C. This normalization was implemented to increase the contrast and render the images more legible without losing the absolute data. Fig. 3e depicts one thermal RGB float image.
- THERMAL_RGB directory This directory contains all 640 × 480pixel PNG of a given traverse. The THERMAL_RGB images are normalized per image, i.e., absolute values cannot be inferred, but they can serve to better understand how terrain features are captured because local contrasts are generally higher. See Fig. 3d for an example.

#### Bumblebee XB3

The Bumblebee XB3 images are exported as 1280 × 960pixel color PNG images. See Fig. 3f for an example. We name the individual XB3 sensors after their position as seen from the rover’s point of view. I.e., as XB3_LEFT and XB3_RIGHT respectively.

#### Transformations

**Fig. 6:**
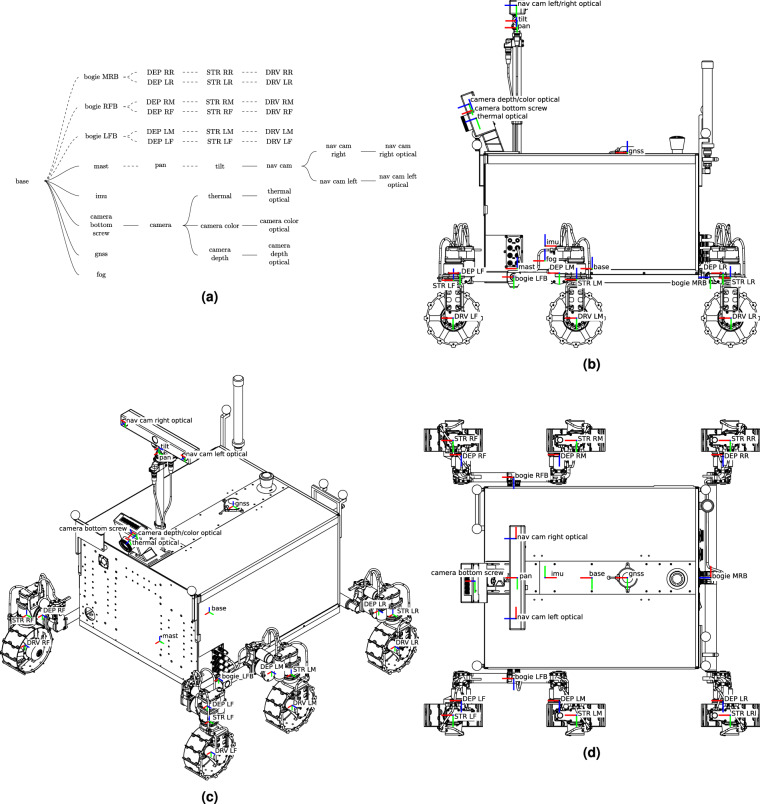
MaRTA’s transformation tree (**a**) and coordinate frames in (**b**) side view, (**c**) 3/4 view, and (**d**) top view. In the transformation tree, dashed lines represent dynamic transformations while solid lines represent static transformations. In the coordinate frame figures, red, green, and blue represent the X, Y, and Z axes respectively.

MaRTA follows the ISO 8855 convention for vehicle coordinate systems, i.e., in MaRTA’s reference frame, *x* is pointing forwards, *y* leftwards, and *z* upwards. The base frame is located in the geometrical center of the body chassis for *x* and *y* (Fig. 6d) and at the chassis bottom plate in *z* (Fig. 6b). In the global, or map, frame, *x* points East-wards, *y* North-wards, and *z* upwards.

MaRTA’s coordinate frames can be seen in Fig. 6b through Fig. 6d. Note that some intermediate coordinate frames are not displayed to obtain a clearer overview. Instead, we opted to visualize the starting frame (base) and leaves (e.g., nav cam left optical) as well as key coordinate frames (mast, gnss, etc.). Figure 6a shows an overview of the entire transformation tree and indicates which transformations are static and which are dynamic. All coordinate frames follow the right-hand convention, the x-axis generally points forward, i.e., in the direction of rover travel, except for optical frames, which follow the camera convention of pointing their z-axis forward, x to the right, and y down. The link names are the same between the static transformations, the dynamic transformations, this paper, and the rosbags. The (F/T) sensors have the same coordinate frames as the corresponding steering joints (STR*), except on the right-hand side: Here, the (F/T) coordinate frames are rotated 180 ° around their *z* axis, resulting in their *x* axis pointing backward, *y* away from the rover, and *z* upwards.

- TF_STATIC.csv Static transformations are saved in each traverse’s TF_STATIC.csv file (Table 2). They comprise the spatial relationship between all fixed components, mainly within the rover’s cuboid chassis plus the sensor assembly at the front. Namely, these are the base link as the transformation tree root, the bottom of the mast, the GNSS antenna, the FOG, IMU, camera box assembly, down to each optical frame within Thermal Camera, Realsense RGB, and Realsense Depth. The last static transformation goes from the tilt unit atop the rover mast to the BBX3 stereo camera and from there to the corresponding optical frames. Note that the IMU is mounted backward.
- TF.csv The dynamic transformations comprise all moving joints of the rover. These are the passive bogies, active motor joints in the six legs, the six wheels, and the PTU. Rotations occur around a frame’s z-axis. One can see this in the wheel driving, steering, and deployment motors (DRV*, STR*, DEP* respectively), the bogies (bogie*), as well as the PTU’s pan and tilt coordinate frames. The format of the dynamic transformations export, i.e., TF.csv, is as follows: Each line describes one transformation via a timestamp followed by the link’s name, its child link, the translation [m] along the three axes and finally the rotation along the three axes expressed as a quaternion.
- TF_CORRECTED_REAR_BOGIE.csv As the name suggests, TF_CORRECTED_REAR_BOGIE.csv contains the same information as TF.csv, except that the rows containing the rear bogie transformations contain more sensible values. The dedicated section in the data validation section details how and why the modified values are computed.

**Table 2:** Static transformations. Translations are provided in meters.

Frame ID	Child Frame ID	*T* = (*T*_*X*_, *T*_*Y*_, *T*_*Z*_)	*Q* = (*Q*_*X*_, *Q*_*Y*_, *Q*_*Z*_, *Q*_*W*_)
base	mast	(0.187, 0, 0)	(0, 0, 0, 1)
mast	fog	(−0.05441, 0, 0.055314)	(0, 0, 0, 1)
mast	gnss	(−0.290212, 0, 0.318500)	(0, 0, 0, 1)
mast	imu	(−0.05175, 0.00326, 0.09974)	(0, 0, 1, 0)
mast	camera bottom screw	(0.11876, 0.01213, 0.43947)	(0, 0.173648, 0, 0.984808)
camera bottom screw	camera	(0.0106, 0.0175, 0.0125)	(0, 0, 0, 1)
camera	camera depth	(0, 0, 0)	(0, 0, 0, 1)
camera depth	camera depth optical	(0, 0, 0)	(−0.5, 0.5, −0.5, 0.5)
camera	camera color	(−0.000173, 0.014993, 0.000084)	(0.006413, 0.000537, −0.00352, 0.999973)
camera color	camera color optical	(0, 0, 0)	(−0.5, 0.5, −0.5, 0.5)
camera	thermal RGB	(−0.018, −0.029, −0.047160)	(0, 0, 0, 1)
thermal RGB	thermal RGB optical	(0, 0, 0)	(0.5, −0.5, 0.5, −0.5)
tilt	nav cam	(0, −0.015, 0.0095)	(0.707107, 0, 0, 0.707107)
nav cam	nav cam left	(0, 0.12, 0.0185)	(0, 0, 0, 1)
nav cam left	nav cam left optical	(0, 0, 0)	(0.5, −0.5, 0.5, −0.5)
nav cam	nav cam right	(0, −0.12, 0.0185)	(0, 0, 0, 1)
nav cam right	nav cam right optical	(0, 0, 0)	(0.5, −0.5, 0.5, −0.5)

#### Laser-Induced Breakdown Spectroscopy (LIBS)

**Fig. 7:**
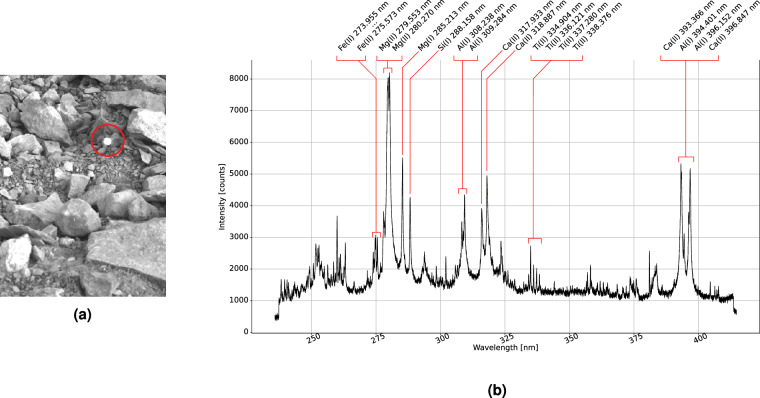
Photo of measurement location L16 (**a**) and its annotated spectrum (**b**). The bright spot in the photo is the laser-induced plasma which marks the point of analysis. The annotations only serve this paper but are not present in the dataset.

The libs_measurements.csv lists the following information about each recorded spectrum in columnar form: (1) Spectrum identifier, (2) location number, (3) longitude [deg E], (4) latitude [deg N], (5) date [YYYY-MM-DD], (6) local time [HH-MM-SS], (7) path to the CSV containing the spectrum, and (8) photo of the spectrum sample [.bmp]. X/Y/Z.bmp is spectrum number *Z* of the *Y*th acquisition at location number *X*. The corresponding spectrum data can be found in column *Z* of X/Y/spectra.csv. Figure 7 shows a measurement at location L16 and its spectrum in which we can observe a high contribution of iron and magnesium.

#### Drone map

The geo-referenced drone map of the test area is available in the following formats:

- mesh A simplified, textured 3D model of the environment.
- point cloud A high-resolution, densified point cloud as binary .ply and .xyz, both containing map color information.
- mosaic A high-resolution ortho-rectified mosaic .tif.
- Digital Surface Model (DSM) A high-resolution .tif containing altitude information.

#### Weather station

The weather station data consists of one CSV per day, each containing five columns: (1) Local time [HH:MM:SS], (2) Solar radiation [W/m^2^], (3) Temperature [°C], (4) Humidity [%], and (5) Pressure [hPa].

## Technical Validation

While recording the data in the field, sensor readings were visualized to allow for sanity checks and stop recordings in case of anomalies. Only traverses with continuous GNSS position corrections through RTK were considered.

During the field tests, issues were discovered with the PTU and rear bogie sensor readings, for which workarounds were devised on the spot. This section reports on the dataset’s reliability, the above-mentioned issues, explains how calibrations were performed, and elaborates how corrections were applied to the dataset. Note that all raw data is available in addition to the corrected data .

### GNSS covariance

The reported Position_Covariance_Type of 2 indicates that the diagonal of the covariance matrix is known, but not the rest of the matrix. The other possible values signify: (0) Unknown, (1) Approximated, (2) Diagonal Known, and (3) Known. In the GNSS covariance matrix (equation (3)), this is indicated by question marks in the unspecified fields.

3 $${\rm{GNSS\; Covariance\; Matrix}}\,=\left[\begin{array}{ccc}\,{\rm{Var}}\,(x) & \,{\rm{Cov}}\,(x,y) & \,{\rm{Cov}}\,(x,z)\\ \,{\rm{Cov}}\,(y,x) & \,{\rm{Var}}\,(y) & \,{\rm{Cov}}\,(y,z)\\ \,{\rm{Cov}}\,(z,x) & \,{\rm{Cov}}\,(z,y) & \,{\rm{Var}}\,(z)\end{array}\right]=\left[\begin{array}{ccc}0.000196 & ? & ?\\ ? & 0.000196 & ?\\ ? & ? & 0.000256\end{array}\right]$$

The RTK receiver reported high-fidelity position corrections throughout all traverses. In the few instances where this was no longer the case, the traverse was immediately stopped. Additionally, the dataset provides an overview plot of all individual traverses to help assess the GNSS accuracy and highlights all areas of traverses in yellow circles in which we spotted positional errors when comparing to camera images. The recommended timestamps in Table 3 take this into account by rejecting GNSS jumps.

**Table 3:** Start and end time recommendations for each traverse.

Recording	Traverse start	Traverse end	FOG heading offset [*rad*]	Cameras
2023-07-20 18-12-05	18:13:32 (1689869612)	18:35:04 (1689870904)	1.2881	All
2023-07-20 19-12-27	19:13:04 (1689873184)	19:14:51 (1689873291)	2.3	All
2023-07-20 20-01-38	20:02:57 (1689876177)	20:22:23 (1689877343)	−3.2501	None
2023-07-21 12-38-15	12:38:30 (1689935910)	12:52:05 (1689936725)	−1.8	All
2023-07-21 12-58-11	12:58:13 (1689937093)	13:42:05 (1689939725)	4.5	All
2023-07-21 13-43-00	13:43:01 (1689939781)	13:54:16 (1689940456)	-1.723	All
2023-07-21 13-59-14	13:59:15 (1689940755)	14:04:57 (1689941097)	4.5148	All
2023-07-21 14-08-29	14:08:33 (1689941313)	14:38:53 (1689943133)	−1.7145	All
2023-07-21 14-44-56	14:44:57 (1689943497)	14:49:13 (1689943753)	4.4302	All
2023-07-21 14-51-07	14:51:08 (1689943868)	15:03:32 (1689944612)	-1.8	Only XB3
2023-07-21 17-07-00	17:07:03 (1689952023)	17:31:25 (1689953485)	0.98	All
2023-07-21 17-34-18	17:34:21 (1689953661)	17:43:42 (1689954222)	1.82	All
2023-07-21 17-45-42	17:45:43 (1689954343)	18:11:33 (1689955893)	−4.4474	All
2023-07-22 13-00-57	13:01:28 (1690023688)	13:09:40 (1690024180)	0.34	All
2023-07-22 13-28-55	13:29:21 (1690025361)	13:50:52 (1690026652)	2.06	No XB3
2023-07-22 14-18-23	14:18:47 (1690028327)	14:53:33 (1690030413)	2.165	No XB3
2023-07-22 16-24-27	16:24:50 (1690035890)	17:16:55 (1690039015)	0.2374	All
2023-07-22 17-18-36	17:18:56 (1690039136)	17:20:15 (1690039215)	0.28	All
2023-07-22 17-31-58	17:32:15 (1690039935)	17:34:51 (1690040091)	0.28	All
2023-07-22 17-38-50	17:39:07 (1690040347)	17:54:31 (1690041271)	0.5	All
2023-07-23 11-23-18	11:23:36 (1690104216)	11:44:50 (1690105490)	−1.16	All
2023-07-23 11-52-09	11:52:28 (1690105948)	12:19:39 (1690107579)	−1.11	All
2023-07-23 12-52-39	12:52:05 (1690109585)	13:04:07 (1690110247)	−1.25	All
2023-07-23 13-05-11	13:05:34 (1690110334)	13:18:51 (1690111131)	−1.1949	All

Times are also provided as epochs in seconds in parentheses. Choosing the heading offset presented in the last column provides the poses in FOG_CORRECTED.csv.

### Rover heading

**Fig. 8:**
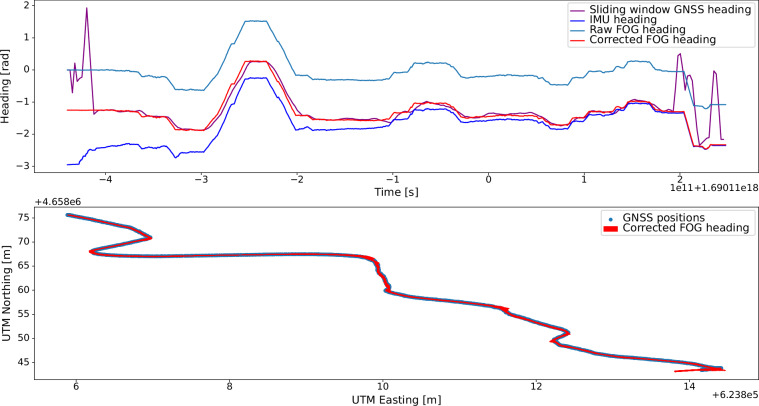
Heading validation and FOG offset correction in the dataset.

The FOG records its heading relative to its starting orientation but it is not aligned to a cardinal direction. The values have been corrected by adding an offset to ensure that an orientation of 0 corresponds to the rover facing East. The heading offsets were chosen individually for each traverse. For completeness, they are also listed in Table 3. A plot such as Fig. 8 of the individual traverses with the recommended headings can be found in the respective traverse directory.

The rover’s heading may be computed by interested parties themselves from the raw values (provided in FOG.csv), or the recommended offset-corrected headings may be used (provided in FOG_CORRECTED.csv).

### Force and torque sensor corrections

**Fig. 9:**
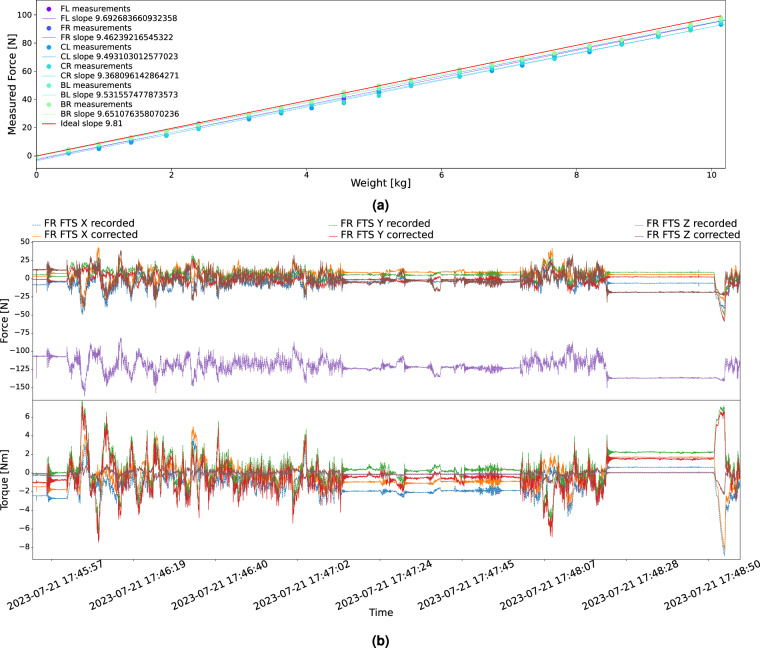
(F/T) sensor correction for slope and offset. Plot (**a**) displays the (F/T) slopes and (**b**) shows the sensor corrections for a 3 min sample. Notice that in (**b**), the *z*-axis (dashed purple line) is corrected from its original range of ca. −150 to −75N to a range of ca. −45 to 30N (solid brown line). Due to the natural terrain the starting values and the average values of the readings are unlikely to be zero.

The (F/T) sensors come pre-calibrated from the manufacturer. However, since they read analog signals and the cables have been reworked, the absolute readings are no longer calibrated. To address this issue before the data collection, the linear behavior of each sensor was verified and the individual slopes were recorded. The verification involved placing known weights on each sensor and recording the (F/T) response. To account for any imperfect leveling, the forces of all three axes were combined. Increasing the weight from 0 kg to 1 kg, the expected force would increase by 9.81 N. Figure 9a shows the linear regression results, where it can be seen how close the recorded values are to the expected slope. Both forces and torques are derived from six channels of strain gauge voltages. We assume that all channels are affected similarly by the altered cable and therefore the same correction factor that is applied to a sensor’s forces is also applied to its torques.

Next, the base or offset of the sensors was recorded. The rover was placed on a flat ground in its field test configuration and a series of (F/T) values were recorded. With base and slope known, the recorded forces and torques could be corrected. Each traverse directory contains the raw values, the corrected values in a separate CSV, and the comparison between raw and corrected values in an SVG such as Fig. 9b.

### Pan and Tilt Unit (PTU) zeroing

The PTU is used to point the Bumblebee XB3 stereo camera on MaRTA’s mast towards the ground ahead of the rover. It uses potentiometers as absolute position sensors, which are prone to drift, which was noticeable during the field tests. To cope with this, we zeroed the PTU at least once per day before recording the day’s camera calibration images and thereby before the day’s first traverse. This was repeated in case we noticed a deviation between the reported and actual angles.

### Rear bogie decalibration

**Fig. 10:**
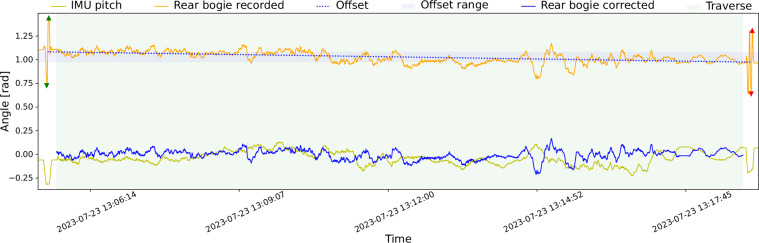
Bogie offset estimation.

During the field testing activities with MaRTA, we noticed that the sensor readings of the rear bogie position were not consistent. After a longer recording period, it would show a highly inclined bogie position, which was not in line with the actual configuration. A first analysis of the problem pointed towards an encoder issue, which we were unable to resolve in the field. As a workaround, we introduced a calibration procedure at the beginning and end of each test run: we moved the rear bogie into both end-stop positions and recorded the extreme sensor values. Under the assumption that the encoder readings decalibrate slowly and nearly linearly, this would allow to correct the bogie position while post-processing the dataset. An example can be seen in Fig. 10.

We acknowledge that these corrections may not be perfect, which is why we also provide the raw, unaltered measurements. However, the corrected values are more sensible and could serve as a useful initial assessment of a specific traverse, particularly for those interested in the bogie transformations, or simply for more appealing full-rover visualizations.

### Camera calibration and sensor alignment

On each day of the field test, we captured calibration images of an April tag calibration board using the Bumblebee XB3 cameras and the Realsense RGB camera. The results of the corresponding multi-camera calibrations, conducted with Kalibr, can be downloaded as part of the dataset.

### Weather station

The pyranometer has a resolution of 1 W/m^2^ and an accuracy of ±5 % plus 45 W/m^2^ for every 30 m of cable. In our case, the cable had a length of only 15 cm and is thus negligible.

## Usage Notes

We recommend to treat the timestamps in Table 3 as traverse boundaries. The timestamp recommendations are informed by the rear bogie corrections. The intervals have unobstructed camera images and have sensible positional data. Table 3 also lists the applied FOG offset for each traverse.

The camera images are intentionally left unrectified in the dataset so that users can apply their own rectification or work on the raw images. Multi-camera calibration results obtained via the Open Source software Kalibr are provided in the rover_sensors/calibration directory for reference. These resulting parameters contain the camera intrinsics for image rectification and also contain the extrinsics between the cameras to enable stereo processing and co-registration.

As alluded to in the Background and Summary section, BASEPROD^15^ provides additional data for researchers and engineers working on GNC tasks such as visual odometry thanks to the camera images and GNSS data. Furthermore, sensor fusion techniques can be explored and validated due to the additional availability of the transformation tree information and depth information. Localization and mapping tasks benefit from BASEPROD’s^15^ 3D and orthomosaic terrain maps. The dataset also lends itself to terrain segmentation tasks using a combination of color images, thermal data, and depth information, allowing for the development of algorithms that can distinguish between various analogue terrain types, such as rocky, sandy, dried-up riverbeds, and outcrops. The different modalities, all capturing the terrain ahead from both similar (fixed cameras and thermal camera) and distinct vantage points (mast-mounted camera) with overlapping regions of interest, enable engineers to explore innovative approaches to advance segmentation tasks.

In terms of proprioception, terrain classification can be investigated using the IMU and/or (F/T) sensors. These sensors provide critical data on the physical interaction between the rover and the terrain it traverses. This information can be used for developing algorithms that detect, classify, and adapt to different surface types, thereby increasing the rover’s stability and safety in challenging environments.

The value of the dataset is derived from the length of the traverses, the diverse terrain types, the additional inclusion of uncommon sensors, and the open data formats. As shown in the above list of potential applications, we expect BASEPROD^15^ to not only serve our own research in planetary exploration, but also to be a valuable resource for the broader scientific and engineering community working in this field.

## Data Availability

The delogging and visualization scripts used to create the dataset and this paper from the rosbags can be found on Github (https://github.com/spaceuma/baseprod). All requirements and versions are stated in the git repository’s README.md and requirements.txt.
